# A pharmacist‐physician collaborative care model in chronic kidney disease

**DOI:** 10.1111/jch.14372

**Published:** 2021-10-26

**Authors:** Hanlin Li, Jai Radhakrishnan

**Affiliations:** ^1^ Lead Clinical Pharmacy Manager Ambulatory Care Department of Pharmacy NewYork‐Presbyterian Hospital New York New York USA; ^2^ Professor of Medicine at Columbia University Irving Medical Center Clinical Director Division of Nephrology Columbia University Irving Medical Center/NewYork‐Presbyterian Hospital New York New York USA

**Keywords:** clinical pharmacology, diabetes mellitus, hypertension, outcomes of care, technology

## Abstract

A multidisciplinary approach, including pharmacist interventions, has improved clinical outcomes and reduced economic costs in chronic disease management. To our knowledge, this is the first study to describe the incorporation of pharmacist‐driven medication management with partnering nephrologists while capitalizing on technology to improve outcomes in a Chronic kidney disease (CKD) population. The authors created a collaborative drug therapy management agreement to enhance medication management for hypertension and diabetes mellitus. Our metrics aligned with the Healthcare Effectiveness Data and Information Set (HEDIS) measures. Most referrals to the program met the HEDIS definition of suboptimal disease control (Diabetes: 19/25 and Hypertension: 45/70). In patients with multiple pharmacy visits, achievement of diabetes targets increased from 23% to 67% and hypertension from 29% to 58%. Those who used technology tools were more likely to reach goals. The positive impact of a pharmacist‐physician collaboration and utilization of technology tools present an opportunity to improve care for CKD patients.

## 1 BACKGROUND

Chronic kidney disease (CKD) is a public health concern given its prevalence, associated morbidity, and mortality risk.^1^ A multidisciplinary approach, including pharmacist interventions, has improved clinical outcomes and reduced the financial burden in chronic disease management.^2^ Established data is available mostly for primary care clinics, literature examining CKD patients and effectiveness of such collaboration with specialty practices is limited.^3^, ^4^, ^5^ CKD‐related uncontrolled diabetes mellitus and hypertension are associated with macrovascular and microvascular complications.^6^ Additionally, renal impairment accompanied by a complex CKD medication regimen creates an opportunity for a pharmacist‐physician collaboration to optimize disease management.

Furthermore, telehealth is the newest and preferred blueprint as clinicians continue to leverage technology to deliver medicine.^7^ Audio/video visit models, electronic applications, and remote patient monitoring programs are examples of tools that can enhance disease management and facilitate patient engagement in care.^8^ To our knowledge, this is the first study to describe the experience of incorporating pharmacist‐driven medication management with partnering nephrologists while capitalizing on technology to improve outcomes in a CKD population.

### 1.1 Program description

The pharmacist created a collaborative drug therapy management agreement with nephrologists to optimize medication management for CKD‐associated disease states, including hypertension and diabetes mellitus.^9^ This scope of practice allows nephrologists to refer established patients to the pharmacist for medication adjustment. An established patient is a CKD patient who regularly follows with the practice. The visits with the pharmacist enhance the existing care under the nephrologists–more frequent follow‐ups with the pharmacist in between physician visits allowed for timely goal attainment.

During the appointment, the pharmacist collected subjective information around medication adherence, access, medication effectiveness (eg, home blood pressure readings), medication tolerance, and lifestyle practices such as diet. Then reviewed objective laboratory parameters and formulated an assessment and plan, including an adjustment in medications, order of laboratories, triage to other services as appropriate. When available, the pharmacist also engaged the patient's family members or caregivers during the encounter. Visits were primarily in‐person before the coronavirus pandemic but have seamlessly transitioned to telehealth without disrupting care.

Another unique aspect of our care model is the use of technology resources to enhance patient engagement. The remote patient monitoring program for CKD targeted those with uncontrolled hypertension. We provided patients with a blood pressure monitor kit connected to a tablet device to capture vitals at home. Data was automatically transmitted and stored within the electronic medical record (EMR) for the team to review regularly. Patients were continuously monitored–any reading outside of the pre‐identified parameters would trigger an alert. Alternatively, patients with technology access who preferred to use their own devices could upload home readings directly onto the patient portal part of the EMR system. The pharmacist reviewed the patient's home log during the visit for those who opted out of either tool.

Our metrics aligned with the Healthcare Effectiveness Data and Information Set (HEDIS) measures, which are benchmarks to evaluate performance.^10^ Uncontrolled hypertension is systolic blood pressure (SBP) greater than 140 mm Hg or diastolic blood pressure (DBP) greater than 90 mm Hg, whereas controlled is when SBP is less than 140 mm Hg and DBP is less than 90 mm Hg.^10^ Guideline recommendations were taken into consideration when developing individualized patient goals.^11^, ^12^ Additionally, inadequate diabetes control has a cutoff of a hemoglobin A1c greater than 9%, and the goal is an A1c less than 8%.^10^ We performed an IRB‐approved, retrospective study to evaluate the program.

### 1.2 Outcome

Our patient characteristics and consult reasons are summarized in Table 1. The pharmacist documented a total of 333 interventions and communicated with the referring nephrologists regularly. Examples included dose adjustment and laboratory monitoring. Patients had a median of three pharmacy visits (range 1–25) over a median follow‐up time of 3 months (range 0.5–17). The frequent follow‐up with the pharmacist and concentrated visits ensured a continuity of care and helped patients to remain engaged for disease management.

**TABLE 1:** Patient characteristics

Age, mean (SD)	69±13.8
Female, *n* (%)	45 (49%)
Male, *n* (%)	47 (51%)
Race, *n* (%)	
White	35 (38%)
Black or African American	18 (20%)
Asian	4 (4%)
Native Hawaiian/Other Pacific Island	1 (1%)
Other	12 (13%)
Declined	21 (23%)
Primary language, *n* (%)	
English	67 (73%)
Spanish	16 (17%)
Chinese	1 (1%)
Other	7 (8%)
Reason(s) for referral, *n* ^a^	
Hypertension	70
Diabetes	25

^a^ *A nephrologist may refer a patient for more than one condition (eg, hypertension and diabetes)*.

**FIGURE 1:**
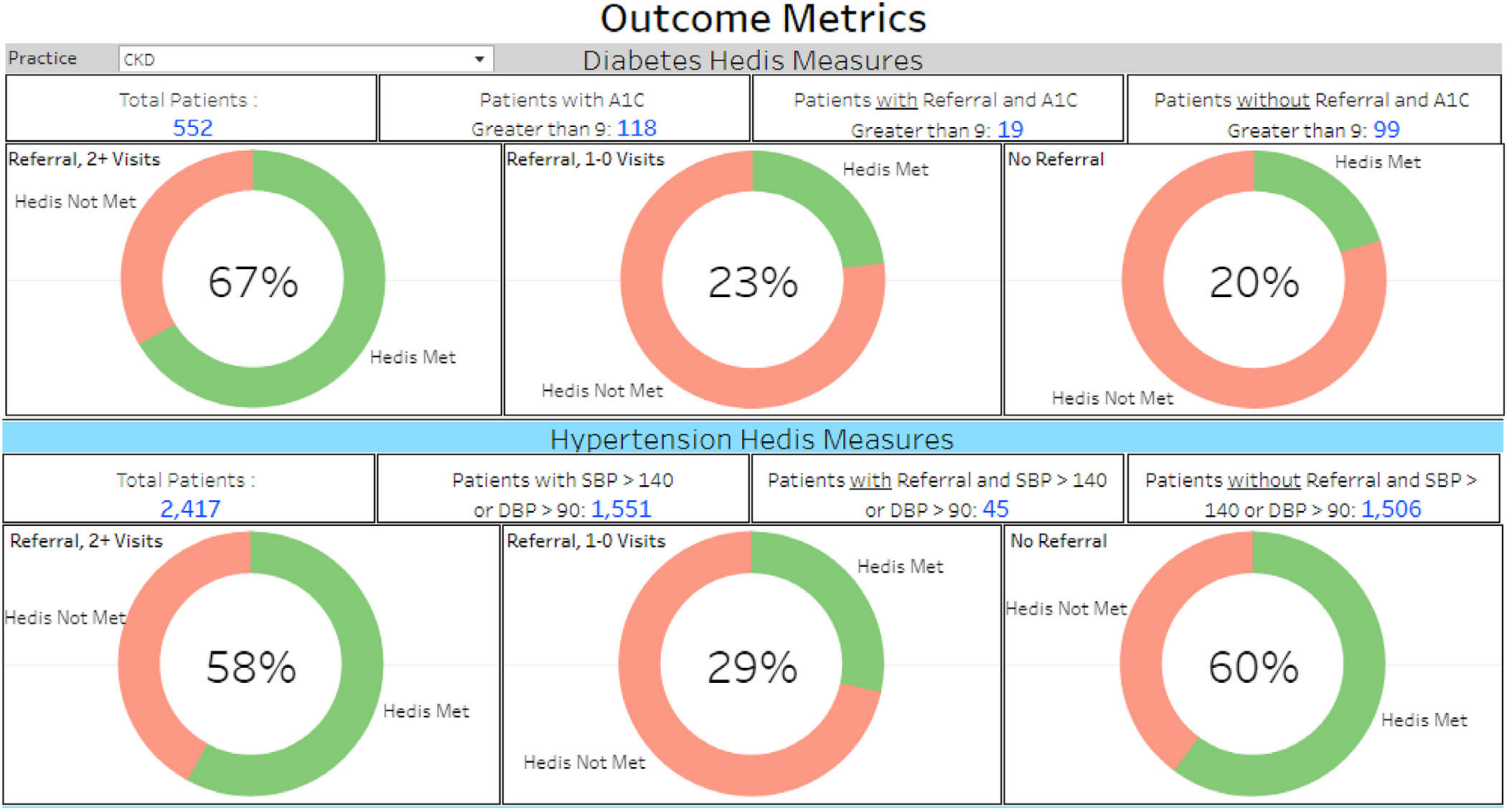
Dashboard outcome metrics. The illustrations are for diabetes and hypertension HEDIS measures target achievement rates. For each disease state, the first diagram shows the % at goal for those with pharmacist referral and seen two or more times; the second diagram shows the % at goal at the time of referral or the initial pharmacy visit; the third diagram shows the % at goal with no pharmacy referrals

We tracked real‐time goal attainment rates in our dashboard to optimize clinic utilization (Figure 1). Most referrals to the program met the HEDIS definition of suboptimal disease control (Diabetes: 19/25 and Hypertension: 45/70). Patients were already on multiple medications at the time of referral. A significant increase in the percentages of patients meeting treatment targets was observed across the disease states. In patients with two or more pharmacy visits, achievement of diabetes targets increased from 23% to 67% and hypertension from 29% to 58%. Further analysis of the data revealed that patients with pharmacist interventions plus a technology tool (eg, utilization of the patient portal) achieved a higher goal attainment rate for hypertension (73% vs. 50%).

## 2 CONCLUSIONS

The positive impact of a pharmacist‐physician collaboration presents an opportunity to optimize care for CKD patients. With the National Kidney Initiative and a focus on a value‐based model, incorporating a team‐based approach is needed to enhance the services and improve outcomes in this vulnerable population.^13^ The success of the pharmacist‐physician collaboration is observed as the role of team members evolved.^14^ Given healthcare complexity continues to increases, it is crucial to recognize the need to synergize efforts to maximize efficiency across the spectrum of care.

We observed that utilizing technology tools was feasible and patients who used such tools were more likely to achieve goals.^15^ Long‐term outcomes, including progression of disease and healthcare utilization, are to be assessed in a prospective, randomized trial to validate the efficacy of pharmacy services and technology. Further analysis is needed to confirm key drivers of patient activation and associated impact on outcomes.

## CONFLICT OF INTEREST

None declared.

## FUNDING

None.

## AUTHOR CONTRIBUTIONS

All authors have reviewed and approved the contents of the manuscript; Hanlin Li and Jai Radhakrishnan implemented the program and carried out the workflow. Hanlin Li analyzed the data and drafted a significant portion of the manuscript. Jai Radhakrishnan supervised the final manuscript.
